# Familiar Forms of Nervous Disease

**Published:** 1890-12

**Authors:** 


Familiar Forms of Nervous Disease. By M. Allen Starr, M. D., Ph. D., Professor of Diseases of the Mind and Nervous System, College of Physicians and Surgeons, New York. With illustrations, diagrams and charts. 8vo; pp. xii.—339. New York: William Wood & Company. 1890.

The work before us, as the author states, is not a treatise upon nervous disease—only a series of clinical studies of the more familiar forms. The data which have been utilized in the preparation of these studies has been selected chiefly from the nervous clinic of the College of Physicians and Surgeons, New York. The object of the work is a commendable one, inasmuch as it places before the medical world the experience of a body of men with a complex and compound series of diseases, the solution of which, clinically and therapeutically, is of great importance. The formulation of general rules in the treatment of such cases must come from large clinics, where cases may be massed and treatment carefully watched. The author, with his able corps of assistants, has chosen typical cases of nervous and mental diseases, and pointed out the indication of medical or surgical treatment, as the case may be, and, whenever possible, has given the results of such treatment.

The first eleven chapters the author devotes to personal study and observation of brain and cord lesions. In reviewing the work accomplished in this department, numerous cases are reported which illustrate the points in discussion—adding materially to its interest and instructiveness. The results of surgical procedure have been, whenever practicable, good.

Chapter XII. is devoted to a clinical study of locomotor ataxia, and the results of treatment. The suspension method, although unfavorably reported upon, still seems to have held its own against any one remedy used. It was employed three months, and then allowed to fall into disuse. Of ten cases treated, four cases have improved, three appear stationary; in the remainder the disease is progressing. One patient has died. We hardly believe that such is proof sufficient of its "inability to relieve the symptoms."

Thirty-one cases of infantile paralysis are reported, well classified, and the important points freely commented upon.

Twenty-three cases of paralysis agitans are reported, and a careful study of them has been made by Dr. Peterson, chief of clinic. In five cases tremor of the head was noted, tending with other observations to question Charcot's statement, that tremor of the head is secondary to that of the body. The treatment of these cases has been either empirical or symptomatic, without any brilliant results.

The chapters on Multiple Neuritis and Paramyoclonus Multiplex by the author are very complete, and are followed by an extended bibliography.

Chapter XVI. is devoted to the study of Chorea, by Dr. Vought; 124 cases were under treatment, of whom three suffered from previous attacks of malaria, twenty-one from rheumatism. Improvement or cure generally took place during the fourth week.

Chapter XVII. is devoted to epilepsy. Tincture of simulo has been employed as a remedial agent in fourteen cases; the drug had the effect of lessening the number of attacks; especially in the grand-mal, but had no effect whatever upon petit-mal.

Chapter XX. treats of the ordinary forms of insanity, under the charge of Dr. Peterson. Seventeen cases were admitted and treated.

In all, some 2,000 cases were under treatment from February, 1888, to January, 1889. Their methodical and careful analysis, although a matter of great labor and time, is of much interest and value, and, like the author, we indulge in the hope that similar works may be published by the large clinics in this country. William Wood & Company have added greatly to the treatise by their excellent typographical work. W. C. K.

